# The temperature‐size rule in *Daphnia magna* across different genetic lines and ontogenetic stages: Multiple patterns and mechanisms

**DOI:** 10.1002/ece3.3933

**Published:** 2018-03-13

**Authors:** K. Natan Hoefnagel, E. H. J. (Lisenka) de Vries, Eelke Jongejans, Wilco C. E. P. Verberk

**Affiliations:** ^1^ Department of Animal Ecology and Physiology Radboud University Nijmegen The Netherlands

**Keywords:** body size, *Daphnia magna*, development, genetic line, growth, resource allocation

## Abstract

Ectotherms tend to grow faster, but reach a smaller size when reared under warmer conditions. This temperature‐size rule (TSR) is a widespread phenomenon. Despite the generality of this pattern, no general explanation has been found. We therefore tested the relative importance of two proposed mechanisms for the TSR: (1) a stronger increase in development rate relative to growth rate at higher temperatures, which would cause a smaller size at maturity, and (2) resource limitation placing stronger constraints on growth in large individuals at higher temperatures, which would cause problems with attaining a large size in warm conditions. We raised *Daphnia magna* at eight temperatures to assess their size at maturity, asymptotic size, and size of their offspring. We used three clonal lines that differed in asymptotic size and growth rate. A resource allocation model was developed and fitted to our empirical data to explore the effect of both mechanisms for the TSR. The genetic lines of *D. magna* showed different temperature dependence of growth and development rates resulting in different responses for size at maturity. Also, at warm temperatures, growth was constrained in large, but not in small individuals. The resource allocation model could fit these empirical data well. Based on our empirical results and model explorations, the TSR of *D. magna* at maturity is best explained by a stronger increase in development rate relative to growth rate at high temperature, and the TSR at asymptotic size is best explained by a size‐dependent and temperature‐dependent constraint on growth, although resource limitation could also affect size at maturity. In conclusion, the TSR can take different forms for offspring size, size at maturity, and asymptotic size and each form can arise from its own mechanism, which could be an essential step toward finding a solution to this century‐old puzzle.

## 1 INTRODUCTION

The temperature‐size rule (TSR; Atkinson, 1994) describes the widespread phenomenon that ectotherms reared under warm conditions grow faster, but to a smaller size‐at‐age when compared to conspecifics grown under colder conditions. The TSR is often assessed at size at maturity or first reproduction (Fischer & Fiedler, 2002), but studies have found similar thermal responses for asymptotic size (Martinez‐Jeronimo, 2012) and size of offspring (Atkinson, Morley, Weetman, & Hughes, 2001). The TSR constitutes a life‐history puzzle (Atkinson & Sibly, 1997), since one would expect faster‐growing animals to mature at a larger size, given the advantages of large size (i.e., increased fecundity, competitive advantage). Indeed, when growth rates are enhanced due to more favorable food conditions, fast growth results in a larger size (Kindlmann, Dixon, & Dostalkova, 2001; Yasuda, Miyamoto, Fujiwara, Yamamoto, & Yusa, 2016). Recognizing that both extreme cold and extreme heat could impair growth and reduce body size, Atkinson (1994) explicitly specified the condition that the TSR should be evaluated across a thermal range of nonextreme temperatures (see also Walczyńska, Kiełbasa, & Sobczyk, 2016).

Several physiological mechanisms have been proposed to underlie the TSR, but it remains difficult for a single explanation to encompass all forms of the TSR and at the same time also explain notable exceptions to the TSR (see, e.g., Atkinson, 1995; Fischer & Fiedler, 2002; Horne, Hirst, & Atkinson, 2015; Walters & Hassall, 2006). It is becoming clear that it is unlikely that a general explanation exists (Angilletta & Dunham, 2003; Angilletta, Steury, & Sears, 2004; Forster, Hirst, & Woodward, 2011). Instead, different mechanisms may be applicable to different animal groups (Forster et al. 2011) or situations (Calboli, Gilchrist, & Partridge, 2003). Here, we focus on two explanations that have been proposed to explain the temperature‐size rule. They are both based on physiological rates but differ in their applicability to either size at maturity or asymptotic size.

The first explanation builds on the notion that growth and differentiation (development) are two distinct processes that together govern size and age at maturity (Smith‐Gill & Berven, 1979; van der Have & de Jong, 1996) and argues that development has a steeper thermal reaction norm than growth. Consequently, resource allocation to development increases relative to allocation to growth at higher temperatures, advancing maturity to a younger age and a smaller size under high temperatures. This explanation, however, does not make predictions about asymptotic size, since in many cases asymptotic size is reached long after maturity is reached. Several recent studies have focussed on differences in thermal dependencies of physiological rates as a proximate explanation for the TSR, and these have mainly taken an ontogenetic perspective. For example, Forster and Hirst (2012) found that temperature‐size responses were different or even reversed in certain developmental stages and generally were stronger at later developmental stages of the brine shrimp *Artemia franciscana*. Examining size responses to temperature at different life stages may therefore be a key to better understand how the TSR arises (Forster et al. 2011).

The second explanation focusses on deceleration of growth at the end of the growth trajectory. This deceleration should occur at a smaller size in warm conditions in order to generate the TSR. von Bertalanffy (1960) formalized this explanation in a model where decelerating growth arises from temperature‐dependent resource limitations rather than temperature‐dependent allocation of resources that feature in the first explanation. In his theory, maximum size is reached when anabolism equals catabolism (von Bertalanffy, 1934). Here, catabolism is taken to equal maintenance rates, since maintenance is the process that counteracts catabolism, and anabolism is assumed to be equivalent to resource uptake. Perrin (1995) realized that temperature mainly increases maintenance metabolism, whereas environmental food availability mostly increases resource uptake. As a result, catabolism equals anabolism at a smaller size when the temperature is higher, but at a larger size when food availability is higher. While the relevant resource for von Bertalanffy's model could be food or oxygen, it was suggested that it is primarily oxygen that limits growth in fish due to limits in gill size (Pauly, 1981) although oxygen limitation may also arise at different levels of biological organization (Atkinson et al. 2006). In support of a role of oxygen in setting body size limits, we previously showed that the TSR in a freshwater isopod crustacean was manifested most strongly under hypoxic conditions, whereas hyperoxia could reverse the TSR (Hoefnagel & Verberk, 2015). Recent studies have also highlighted the potential role of oxygen in setting body size limits (DeLong et al., 2017; van Rijn, Buba, DeLong, Kiflawi, & Belmaker, 2017; Walczyńska & Sobczyk, 2017) shifting the focus toward explanations for a TSR to asymptotic size. von Bertalanffy's model provides an explanation for smaller asymptotic size in warmer environments, but does not make explicit predictions about the effect of temperature on size at maturity. Indeed, since maturity is the end point of ontogenetic development and asymptotic size is the end point of somatic growth, the mechanisms that govern the thermal plasticity in body size at maturity may differ from those that govern asymptotic size.

Both temperature‐dependent allocation of resources (explanation 1) and temperature‐dependent resource limitations (explanation 2) can explain the TSR, and they are not mutually exclusive, since they are based on different physiological rates and focus on different life‐history stages. Thus, support for one mechanism cannot be used as evidence against another. Here we evaluate the TSR across different life stages (size at maturity, asymptotic size, offspring size) in three genotypes of *Daphnia magna* (Straus, 1820) at eight different temperatures and combine empirical data with a resource allocation model. To evaluate support for different mechanisms, we investigate the thermal dependency of rates (growth and development) in each genotype and we investigate size dependency of these thermal reaction norms. We incorporated both thermal dependency of resource allocation and resource limitation into a single resource allocation model, and by “switching on or off” one or both of these mechanisms, we can explore whether either is sufficient to explain the classic temperature‐size rule or that some combination of these mechanisms is needed. We then fitted the model to empirically collected data. Thus, our threefold aim is to (1) document the temperature effects on body size during different life stages in *Daphnia magna*; (2) establish the thermal reaction norms for growth and development in different genotypes and in animals of different size and evaluate how such differences can explain differences in the TSR; and (3) disentangle the two explanations using a resource allocation model fitted to empirical data.

We hypothesize that development rate increases faster with temperature than growth rate does, resulting in younger age and smaller size at maturity at higher temperatures and that the two explanations each have their own specific domain of applicability. We also expect differences in body size between rearing temperatures to be larger for asymptotic size than for those at maturity, because growth trajectories will have had more time to diverge.

## 2 MATERIALS AND METHODS

### 2.1 Collection and maintenance of animals

*Daphnia magna* is a small cladoceran crustacean that inhabits freshwater habitats. Its short life cycle and abundance have made this species a popular species for studies on life‐history and energy budgets (Martínez‐Jerónimo, Villaseñor, Rios, & Espinosa, 1994). Resting eggs of *Daphnia magna* were collected from a small lake in Hilversum, The Netherlands, in spring 2014. These resting eggs are produced after sexual reproduction with hatchlings being genetically different from each other (Robinson, Wares, & Drake, 2013). Resting eggs were kept at continuous darkness and 4°C for at least 3 months before they were hatched in the laboratory to give rise to different genetic lines of which two were used in this study. These two genetic lines were designated lines D and E. Newly produced clonal neonates from these lines were separated until 5–10 vials per genetic line contained offspring of that line. A third genetic line was obtained from a laboratory at Wageningen University, The Netherlands, where it had been kept at room temperature for 15 years since collection from Lake Zwemlust, The Netherlands (Lürling & Tolman, 2010). This laboratory line was designated line C. The stock culture of *D. magna* in our laboratory was kept at a constant temperature of 10°C and 16:8 hr light:dark. Fourteen days before the onset of the experiment (May 2015), five juveniles of each of the three genetic lines were separated into 80‐ml glass vials with Dutch standard water (DSW, 200 mg/L CaCl_2_.2H_2_O, 180 mg/L MgSO_4_.7H_2_O, 100 mg/L NaHCO_3_, 20 mg/L KHCO_3_; NEN 1980) and kept at 20°C. Offspring from these mothers’ second clutch onward were used for the actual experiment in accordance with OECD guidelines (Dufresne & Hebert, 1998; OECD/OCDE 2012) and individually and randomly assigned to one of the eight temperature treatments (10, 15, 18, 20, 23, 26, 28, 30°C). At least five individuals from each of the three genetic lines were placed in each temperature, and individuals that died within the first seven days were replaced resulting in a total of 216 individuals in the experiment. Survival, growth, development, and reproduction were monitored three times per week for each individual from birth to death. The medium consisted of 1.6 × 10^5^ cells/ml (McKee & Ebert, 1996; Algal diet 1800) suspended in DSW and was replaced at each measurement to ensure ad libitum food conditions.

### 2.2 Measurement of physiological rates

Length of each individual *D. magna* was measured to the nearest 0.04 mm three times per week using a stereo microscope with 25 times magnification. The frequency of measurement caused an uncertainty in age estimates of approximately 1 day. Length was measured in live animals from the middle of the eye to the base of the caudal spine following Chopelet, Blier, and Dufresne (2008). Movement of the eye and contraction of the body were sometimes observed to cause a small (maximum 0.12 mm) inaccuracy in the length measurement. Width and thickness of *D. magna* were measured for a representative set of animals to derive an equation for converting length into volume (Equation (1)). We assumed a constant weight‐to‐volume ratio for *D. magna*, so that volume could be used as a proxy for weight. The unit volume was used for statistical analyses and for fitting growth curves. The term body size in this study always refers to (calculated) body volume. Individual *Daphnia* that lived at least 20 days at 10°C or 15°C, or 15 days at 18, 20, 23, 26, 28 or 30°C, were used for characterization of individual growth trajectories (105 in total) and also for fitting the resource allocation model. To estimate asymptotic size, a modified von Bertalanffy growth function for body size (Equation (2)) was fitted to data based on individual *Daphnia* using the nonlinear least square function *nls*() in R. Of the four parameters in Equation (2), Vmax and D were estimated and V_0_ and K were constants.

(1) $$\text{Volume}=0.26\;\times \;{\text{Length}}^{3}$$

(2) $$\text{Body}\;\text{volume}=V_{0}+\left(V_{\max}-V_{0}\right)\times {\left(1-e^{-K\;\times \;D\;\times \;\mathrm{age}}\right)}^{3/D}$$

where length is in mm, *V*_0_ is the volume at age zero, and *V*_max_ is the estimated asymptotic volume. We assumed a 10% increase in length on the first day, which results in a volume at birth of 0.7 × volume at first measurement. *K* is the von Bertalanffy growth parameter (which is not actual growth in the sense of mm/d, but the rate of change in the slope). We fixed (instead of estimated) *K* at a value of 0.046 (see Appendix S1) because of its correlation with *V*_max_ (Pauly, 1979). *D* = 3 × (1 − d) and can be interpreted as a parameter to correct for nonisometric growth of gill surface (Pauly, 1981), where d is the mass scaling exponent for catabolism (von Bertalanffy, 1934). Time is age of the individual in days, with the first measurement at day 1, which was 0–72 hr after release from the mother's brood chamber. The maximum slope of the estimated individual growth curve was used as maximum growth rate (g/d).

The first day when eggs or neonates were observed was taken as the moment of maturity. Animals were considered juvenile before this point and adult beyond this point. Length and age were noted as above. When neonates were observed before eggs were observed (embryonic development occurring between two measurements), age at maturity was approximated by subtracting 2 days from the age at first neonates. Development rate is the inverse of time taken to reach maturity and thus has unit day^−1^.

Rates of growth and development were standardized by expressing them as a percentage of the maximum growth rate (0.56 mm^3^/d) and maximum development rate (0.20 d^−1^) observed in this study, and subsequently compared by calculating the ratio between these standardized rates.

Free‐living neonates were counted at the first measurement day after each release, and lengths of three neonates per clutch were measured. Young were then removed from the experimental unit.

### 2.3 Data analysis

All statistical analyses were performed in R version 3.3.2 (R Core Team 2016). All length measurements were transformed into volume prior to statistical analysis. The maximum slopes of the von Bertalanffy growth function were taken to represent the maximum growth rate of that individual. The effects of temperature, genetic line, and the interaction (whether genetic lines exhibited different thermal responses) were assessed by linear models for each of the response variables (size and age at maturity, size and age at asymptote, offspring size, growth rate, and development rate). In the case of size at maturity and asymptotic size, linear relations with temperature fitted the data best. In the case of neonate size, growth rate, development rate, and the ratio between growth and development rate, thermal effects were curvilinear. Model selection was performed based on AIC values, starting with the full model consisting of first‐, second‐, and third‐order polynomial of temperature and all interactions with genetic line, including genetic line as a main effect for the intercept, and then stepwise reduction in the model until the lowest AIC was reached. The resulting ANOVA (type 3 sum of squares) is presented in tables, and models with higher AIC are given in Appendix S7.

### 2.4 Development of a resource allocation model

We developed a resource allocation model to explore the consequences for each of the two mechanisms for the temperature‐size rule. In this model, resource uptake and allocation vary with temperature independently, unlike other energy budget models such as DEB theory (Kooijman, 2000), which feature a single thermal dependency of all physiological rates. Our model describes growth from birth to asymptotic size and predicts size and age at maturity together with size and age at the asymptote. It is partly based on the von Bertalanffy (1960) growth model and includes equations for the division of resources between growth and maturation.

**Figure 1:**
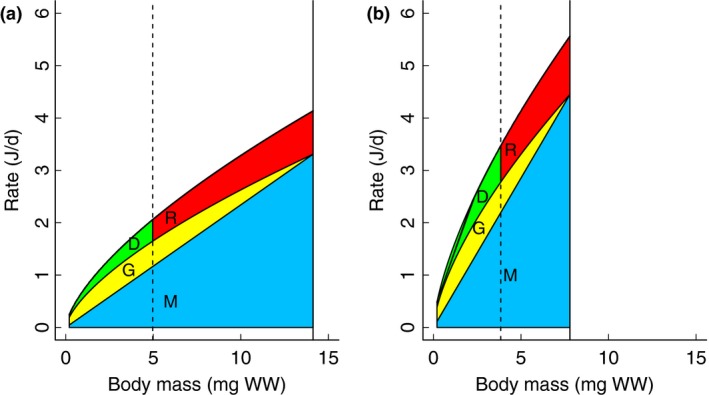
Illustrations of the resource allocation model. Maintenance (M), growth (G), development (D), and reproduction rates (all in J/d) are shown for two constant temperatures (15°C and 25°C). Model parameter values in these illustrations are Q_10,U_ = 2.0, Cu = 0.5, C1 = 0.3, C2 = 1.02, b = 0 (see Equations M1–5), JMat = 3.0, f2 = 0.8. Shaded areas indicate different energy investments: Maintenance (blue) increases isometrically with body size; growth (yellow) is delineated by the line given by f2 × uptake; the difference between Uptake and f2 × Uptake is invested toward Development (green, before maturity) and Reproduction (red, after maturity). The upper black line denotes Uptake rate. Maturity is reached at the vertical dashed line and growth stops at the vertical solid line. Birth occurs at 0.2 mm^3^. Axes are kept constant to illustrate the differences between temperatures. Note also that axes are not log‐transformed

Our model describes five physiological rates expressing energy acquisition and expenditure: resource uptake (U), somatic maintenance (M), with the difference between these two rates allocated to growth (G), development (D), and reproduction (R). Each rate is expressed in relation to wet body mass (*X*; see Figure 1) and in Joules per day. Temperature sensitivity was expressed as Q_1_, which is the factorial increase in a rate at a temperature increase of 1°C. Note that this is not a life‐history optimization model sensu Taylor and Gabriel (1992), but a model to describe resource fluxes in an individual animal. The five physiological rates are given by:

(3) $$\text{Uptake: U}={\text{Q1}}_{U}^{dT}\times \text{Cu}\times X^{d}$$

where *X* is wet body mass, Q1_U_ is the temperature sensitivity of uptake per degree Celsius, and d*T* is the temperature difference with respect to a reference temperature (*T*_ref_ is 10°C in this study). The mass exponent d of anabolism determines how uptake depends on mass and is fixed at **⅔**. Cu is a scaling constant that affects the absolute values of uptake rate.

(4) $$\text{Maintenance: M}={\left(\text{C2}\times {\text{Q1}}_{U}\right)}^{dT}\times \left(\text{C1}\times \text{Cu}\right)\times X^{1}$$

where C1 is a scaling constant for maintenance, C2 × Q1_U_ determines temperature dependence of maintenance. The mass exponent for catabolism (maintenance) was assumed to be 1 (isometry). Maintenance depends on both temperature and body size. A temperature increase of 10°C would cause an increase in physiological rates at any given body size. For example, maintenance would be doubled at a given body size when assuming a Q_10_ of 2 (see Figure 1).

(5) Growth: G=f1×(f2×U‐M)

where f2 denotes that only a certain fraction of the energy consumed is available for growth + maintenance (such that 1 − f2 is available for development and reproduction). This avoids the situation where growth continues until all resources are used for maintenance and no resources are left for reproduction (Czarnoleski & Kozłowski, 1998; Kozłowski, Czarnoleski, & Danko, 2004). And f1 is an allocation function that affects how resources are allocated between growth and development (see eq. (7) below). Parameter f2 was set at 0.8 in this study (see Appendix S2).

(6) Development: D=U−M−G.

Development is assumed to be the only other physiological process that requires resources, thus equalling U–M–G. Energy allocated to development is invested in reproduction after maturity has been reached. Maturity is reached after a fixed maturity threshold of cumulative energy has been invested in Development.

(7) Allocation: f1=1.0−b×dT

where b determines the temperature sensitivity of extra allocation to development (before maturity) or reproduction (after maturity). Allocation does not alter maximum body size, but changes the ratio between energy fluxes to growth and development, thus altering the time needed to reach maximum body size. Only two parameters (b and C2) are required to create scenarios that correspond with each of the two TSR hypotheses, and to fit this model to the shape of the empirical data. Parameters Cu and C1 can be adjusted for vertical scaling to fit the model to the data. These four parameters adjust the five equations which result in temperature dependency and size dependency of all modeled physiological rates (U, M, G, and D/R). After parametrization, the physiological rates can be used to derive the life‐history parameters size at maturity, age at maturity, asymptotic size, and age at (95% of) asymptotic size. Maturity is reached when cumulative development reaches a threshold value (JMat in J), and resources are used for reproduction instead of development after this point. The asymptotic body size is reached when growth equals zero, that is, when the upper boundary of maintenance rate and the line set at f2 × uptake rate intersect. Q_10_ of uptake rate was fixed at 2, which is a common value for biological rates (Daoud, Chabot, Audet, & Lambert, 2007), making Q1_U_ = 2^1/10^ = 1.072. The result of the allocation model is illustrated in Figure 1. See Table 1 for an overview of all parameters and their values.

**Table 1:** Parameters of the resource allocation model. The lower and upper boundaries of relevant parameter ranges are given. The note Fixed indicates that a parameter was not a focal parameter and not allowed to vary. These parameters are fixed on literature values where available. The optimal values for species‐specific parameters were found by brute‐force optimization, these were then fixed at the resulting values to allow finding the optimal values for b and C2 for each genetic line

Para‐meter	Value range	Meaning	Notes
b	−0.01 to 0.03	Temperature dependent allocation to development	Values below −0.01 make growth higher than uptake, values above 0.03 make growth zero. Higher b means more allocation to development
C2	1.0 to 1.04	Temperature dependence of maintenance	Corresponds with Q_10_ for maintenance rate of 2.0–4.0
Cu	0.3 to 0.6	Vertical scaling of rates.	Affects AmaxV and Amat. Species specific.
C1	0.2 to 0.5	Weight dependence of maintenance	Relative to uptake rate. Species specific
JMat	1.6 to 3.0	Bucket for maturity in Joules	Affects size and age at maturity. Species specific
f2	0.8	Fraction to growth + maintenance	Fixed. Model specific
Tref	10	Reference temperature	Fixed. b and C_2_ have no effect at this temperature. Model specific
Q_10,U_	2.0	Q_10_ of uptake	Fixed. General value
d	0.667	Weight exponent of uptake rate	Fixed. General value
J2g	967.5	Converts Joules to grams wet weight	Fixed. Affects growth rate. General value for crustaceans (Wissing & Hasler 1971)

### 2.5 Fitting the resource model to empirical data

The resource allocation model was fitted to the empirical data on the life‐history traits of *Daphnia magna*. Data on size and age at maturity were obtained directly from the experiment, and data on size and age at asymptote were derived from von Bertalanffy growth curves. We only used individuals for which we had values for all four life‐history traits (both from the experiment and from the growth curves), leaving out some individuals that did not reproduce. This resulted in 99 individuals in total, with on average 4 (1–8) individuals per temperature and genetic line. Fitting the resource allocation model was performed by simulating the growth trajectory for certain sets of parameters and then deriving the matching life‐history traits. We used an iterative process starting at *X* = 0.2 and *t* = 1 in which *X*_*t* + 1_ was calculated based on the values for the physiological rates (U, M, G, D) at *X*_*t*_. The energy content of body tissue was set at 4,629 Cal/g dry mass (967.5 J/g wet weight; Wissing & Hasler, 1971). Values for each of the four life‐history traits were subsequently derived from the physiological rates for the different temperatures and compared to the measured values for individual *D. magna*. This whole process was optimized for lowest sum of squared residuals using brute‐force optimization, in which residual sums of squares were calculated for all combinations of parameter values within a certain range. Values for these five parameters were chosen within the ranges indicated in Table 1. First, optimization was performed on all empirical data (lumping the results from different genetic lines). Second, optimal values for the different parameters were derived for each of the three genetic lines separately. In this second optimization, JMat, Cu, and C1 were fixed at the value derived from the optimization based on all data. Thus, genetic lines were only allowed to vary with respect to the parameters that relate to explanation 1 (parameter b, determining the temperature sensitivity of extra allocation to development (before maturity) or reproduction (after maturity)) and explanation 2 (C2, determining the temperature dependence of maintenance).

Sums of squared residuals (SSres) for each life‐history trait were rescaled to prevent life‐history traits with inherently large values (e.g., age at asymptote) from overwhelming life‐history traits with inherently smaller values (e.g., size at asymptote) when minimizing SSres. The rescaling ensures that SSres values are between 0 and 10, where 0 is a perfect fit through the temperature averages and 10 is SStot (the difference between individual data points and a straight horizontal line describing the grand average of a given life‐history trait). Summing SSres over all life‐history traits results in a scale of 0–40, above which fits were neglected.

## 3 RESULTS

Of the 216 *Daphnia magna* individuals used in the experiment, 109 reached maturity. Maximum life span ranged from 146 days at 10°C (mean life span of 90.1 days) to 34 days at 30°C (mean life span of 15.0 days), with genetic line C (the laboratory line) generally living longer than individuals from the other two genetic lines (maximum life span 146 days for genetic line C compared to 115 and 101 days for genetic lines D and E). Of all the individuals that did not reach maturity, 81% died within 3 days after transfer to the experiment and 70% were in temperatures above 25°C. Mortality within 3 days was 16% for genetic line C, 36% for line D, and 55% for line E, such that mortality was much higher in field lines D and E than in laboratory line C. A total of 105 individuals lived long enough to reliably fit a von Bertalanffy growth curve (Equation (2)) through individual size data. Fits of individual von Bertalanffy growth curves were good and resulted in an average *R* ^2^ of .96 (Appendix S1). Observed individual maximum size was generally close to estimated asymptotic size (at 92% ± 20% of asymptotic size).

### 3.1 Effects of temperature on size

**Figure 2:**
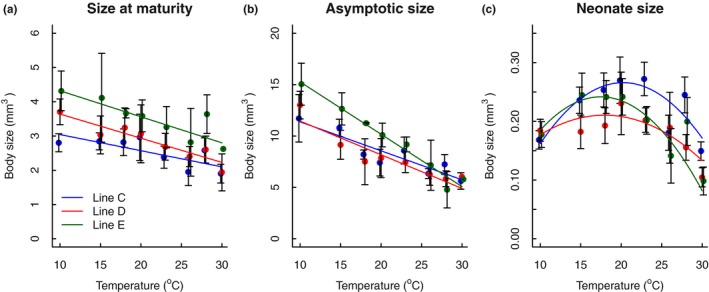
Thermal reaction norms for size in *Daphnia magna*. Size (in mm^3^) at maturity (a) and size at asymptote (b) are given for each temperature and genetic line. Asymptotic body size was estimated using the von Bertalanffy growth function (Equation (2)). Neonate body size (in mm^3^) (c) is averaged across all clutches among individuals within a temperature × genetic line treatment. Colors denote different genetic lines: blue: line C; red: line D; green: line E. Details of the fitted lines are given in Table 1. Error bars denote 95% confidence intervals of the data to indicate statistical differences

Both size at maturity (observed) and asymptotic size (extrapolated from von Bertalanffy growth curves) were negatively affected by temperature, leading to smaller sizes at warmer conditions (maturity: *F*_1,106_ = 8.857, *p* = .004; asymptotic: *F*_1,98_ = 50.838, *p* < .001, Figure 2, Table 2). Offspring size responded nonlinearly to temperature, as indicated by a highly significant effect of temperature squared in the linear model (*F*_1,547_ = 46.84, *p* < .001). Offspring was largest at intermediate temperatures and smaller at both lower and higher temperatures. Genetic lines differed in average size at maturity (*F*_2,106_ = 3.999, *p* = .021) and asymptotic size (*F*_2,98_ = 11.094, *p* < .001) (Table 2), with genetic line E (green) being largest (Figure 2). Importantly, thermal responses, that is, the extent to which temperature reduced size, differed between genetic lines only for asymptotic size (*F*_2,98_ = 6.767, *p* = .002), not for size at maturity (*F*_2,106_ = 0.833, *p* = .437) or neonate size (*F*_2,547_ = 2.818, *p* = .061, Table 2). Repeating these analyses using log‐transformed size yielded similar results (see Appendix S8 for figure and ANOVA tables).

**Table 2:** ANOVA tables (type 3 sum of squares) of linear models for three size measures in *Daphnia magna* based on temperature (T) and genetic line (GenLine), and their interaction. Note the inclusion of temperature squared in the model for neonate size to account for non‐linearity and the different number of observations for the three size measures. ***: p < 0.001; **: p < 0.01; *: p < 0.05

Trait	Source	Sum of squares	*df*	*F*‐value	*p*‐value
Size at maturity	(Intercept)	37.207	1	94.9463	<.001***
*R* ^2^ = .45	Temperature	3.471	1	8.8566	.004**
	GenLine	3.134	2	3.9990	.021*
	T : GenLine	0.653	2	0.8333	.437
	Residuals	41.538	106		
Asymptotic size	(Intercept)	616.91	1	251.7782	<.001***
*R* ^2^ = .69	T	124.56	1	50.8375	<.001***
	GenLine	54.37	2	11.0943	<.001***
	T : GenLine	33.16	2	6.7667	.002**
	Residuals	240.12	98		
Neonate size	(Intercept)	0.0354	1	5.6000	.018*
*R* ^2^ = .16	Temperature	0.2800	1	44.3524	<.001***
	GenLine	0.0283	2	2.2379	.108
	T^2^	0.2958	1	46.8438	<.001***
	T : GenLine	0.0356	2	2.8179	.061
	T^2^ : GenLine	0.0306	2	2.4193	.090
	Residuals	3.4538	547		

### 3.2 Physiological rates associated with the TSR

**Figure 3:**
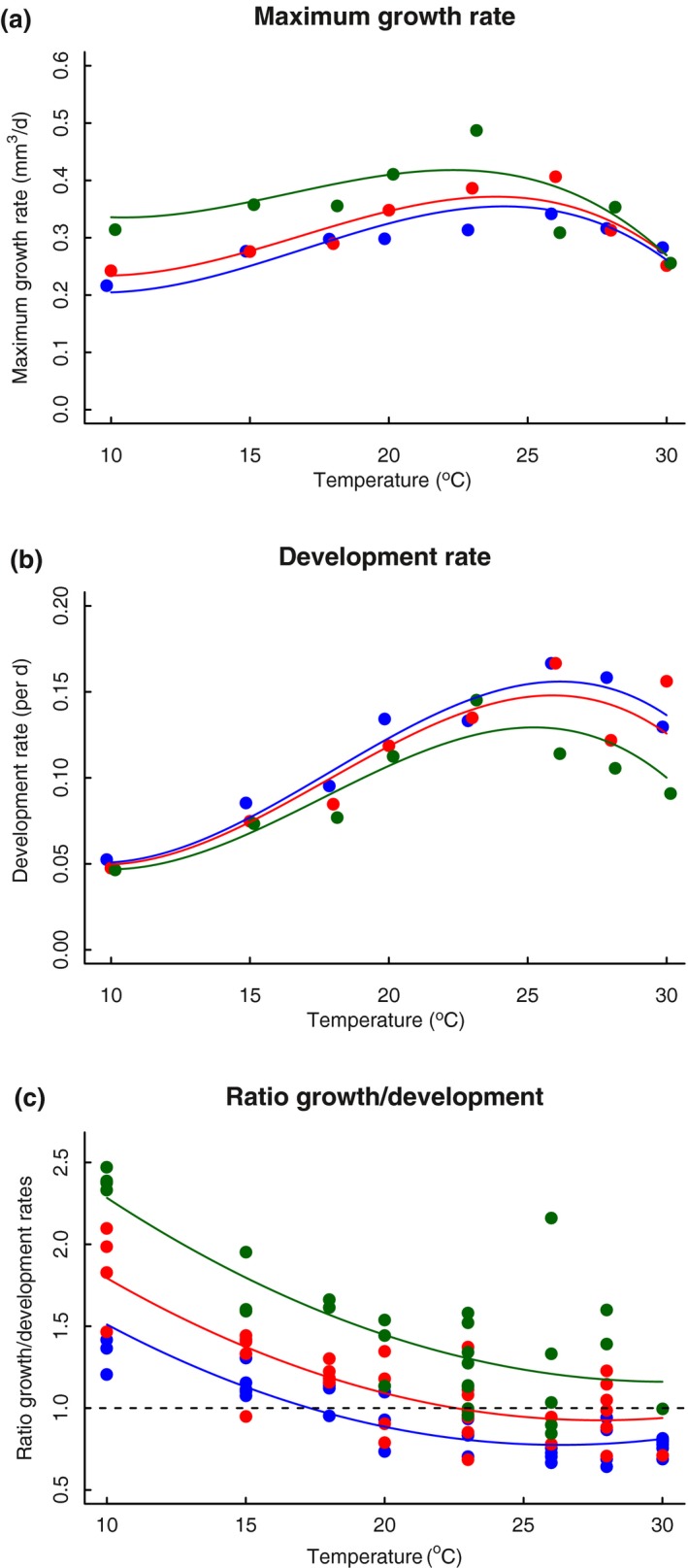
Rates of growth (a) and development (b) in *Daphnia magna*. Average values are given per temperature and genetic line. Maximum growth rate was derived from the slope of individual von Bertalanffy growth curves (Equation (2)). Colors denote different genetic lines: blue: line C; red: line D; green: line E. The rates were expressed as the percentage of their overall maxima to calculate the ratio (c). A ratio of 1 means that rates are at the same percentage of their overall maximum

Both development rate (observed) and maximum growth rate (interpolated from von Bertalanffy growth curves) increase with temperature up to a maximum value before plateauing or decreasing at temperatures beyond 20°C (Figure 3). Thermal responses differed between genetic lines for maximum growth rate (*F*_2,96_ = 7.761, *p* < .001, Table 3) and development rate (*F*_2,106_ = 5.964, *p* = .004, Table 3). Thermal sensitivity for development rates was stronger (approximately threefold increase) than for maximum growth rates (less than a twofold increase; Figure 3), and consequently, maximum growth rates declined relative to development rates with increasing temperature (*F*_1,92_ = 32.070, *p* < .001, Table 3). Genetic line E had relatively high maximum growth rates (Figure 3c), which is concordant with the generally larger size at maturation of this line (Figure 2b).

**Table 3:** ANOVA tables (type 3 sum of squares) of maximum growth rate and development rate in *Daphnia magna*. Note that these analyses were done on different data sets, since not all fully‐grown individuals reproduced . ***: p < 0.001; **: p < 0.01; *: p < 0.05

	Source	Sum of squares	*df*	*F*‐value	*p*‐value
Growth rate	(Intercept)	0.019	1	4.1501	.044*
*R* ^2^ = .42	Temperature	0.013	1	2.7827	.099
	T^2^	0.024	1	5.1128	.026*
	Genetic line	0.073	2	7.7610	<.001***
	T^3^	0.033	1	7.0516	.009**
	T^2^ : GenLine	0.023	2	2.4558	.091
	Residuals	0.451	96		
Development rate	(Intercept)	0.003	1	4.8688	.030*
*R* ^2^ = .60	Genetic line	0.001	2	0.9379	.395
	Temperature	0.004	1	5.2898	.023*
	T^2^	0.007	1	9.7532	.002**
	T^3^	0.008	1	12.3719	<.001***
	T^2^ : Genetic line	0.004	2	2.8618	.062
	Residuals	0.070	104		
Ratio	(Intercept)	4.229	1	99.5206	<.001***
*R* ^2^ = .75	Temperature	1.363	1	32.0705	<.001***
	Genetic line	1.081	2	12.7244	<.001***
	T^2^	0.903	1	21.2516	<.001***
	T : GenLine	0.239	2	2.8085	.065
	Residuals	3.909	92		

**Figure 4:**
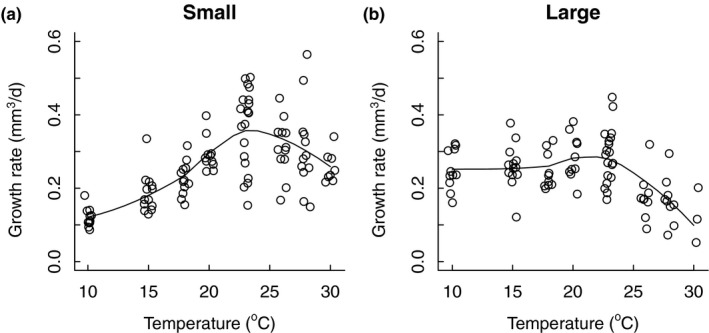
Growth increment (in mm^3^/d) for small (1.2 mm, a) and large (2.6 mm, b) *Daphnia magna*. Data are extracted from individual‐based von Bertalanffy growth curves. Black curves are smoothed averages

Overall, total growth of animals before maturity (juvenile growth) and after maturity (adult growth) correlated positively (*t*_1,83_ = 2.233, *p* = .028, Appendix S6). However, this pattern changed with temperature with total juvenile and adult growth being negatively correlated at the warmest temperatures (*t*_1,83_ = −3.117, *p* = .002, Appendix S6), suggesting that at warmer temperatures growth rates decelerated with increasing body size. Indeed, the body size beyond which growth decelerated (i.e., the body size at which maximum growth rate was achieved) occurred at increasingly smaller sizes with increasing temperatures (*F*_1,98_ = 66.359, *p* < .001, Appendix S6). This effect differed across genetic lines (*F*_2,98_ = 5.990, *p* = .004), and interestingly, temperature caused the strongest decline in size at maximum growth rate in line E which was also the line to show the strongest TSR for final size (Figure 2b). Finally, the thermal sensitivity of growth rates differed markedly between animals of different size, with daily growth peaking at temperatures between 23 and 26°C for small animals (approximately 1.2 mm), but at temperatures between 10 and 23°C for larger animals (approximately 2.6 mm, Figure 4).

### 3.3 Disentangling different physiological mechanisms underlying the TSR

**Figure 5:**
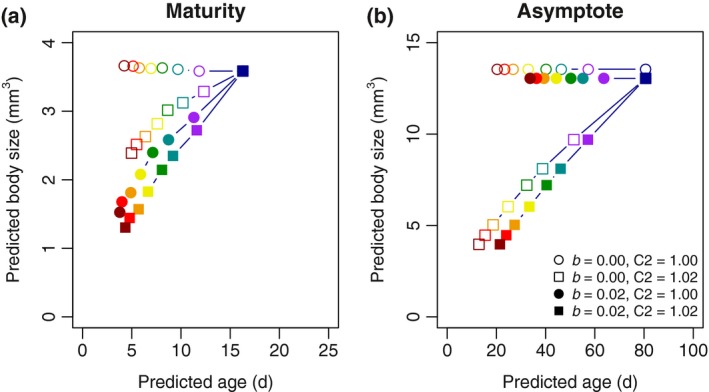
Visualization of the effect of model parameters b (temperature‐dependent extra allocation to development) and C2 (temperature dependence of maintenance rate) on age and size at maturity (left) and age and size at asymptote (right). Colors represent a temperature range from 10°C (dark blue) to 30°C (dark red). Note that the predictions for 10°C remain unchanged as this is the reference temperature in this study. The open circles for asymptotic size (right) coincide with the filled circles, but are offset by 0.5 mm^3^ to improve visual clarity. See Supporting Information for more values for the parameters

The resource allocation model successfully captured the effect that warmer temperatures result in a decreased age at size (i.e., the animals progressed through their ontogeny faster) (Figure 5). Higher temperatures always resulted in decreased age at maturity (dark red symbols always toward the lower values for predicted age), but only resulted in lower predictions for size at maturity when either the allocation fraction (b) is larger than 0 or the thermal sensitivity of maintenance (C2) is larger than 1. Combining a high b with a high C2 did not further decrease predicted age at maturity, but did lead to slightly smaller predicted size at maturity. The faster development at higher temperatures results from factorial increases in both uptake rate and maintenance rate, which lead to increases in the absolute amount of resource that animals can invest in growth and development. Thus, size responses to temperature depended on the specific parameter values. A b higher than 0, indicating that animals preferentially allocate resources to development with increasing temperatures, results in size reductions under warm conditions for size at maturity (Figure 5a), but does not affect asymptotic size (Figure 5b). Increasing C2 in addition to b has minor additional effects on size and age at maturity compared to only increasing b, while size and age at asymptote are chiefly determined by variation in C2.

**Figure 6:**
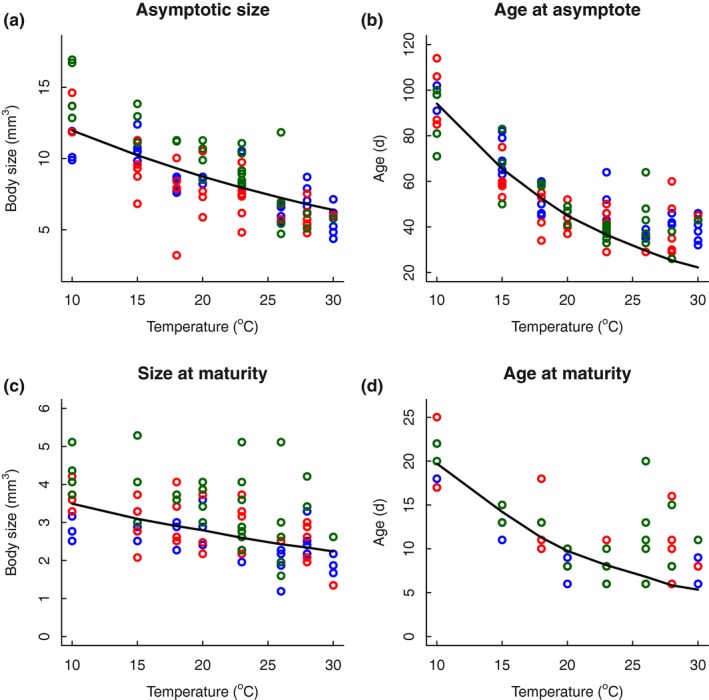
Result of rate model optimization to empirical data on *Daphnia magna*. Life‐history traits were fitted simultaneously. Data are plotted as open circles; colors denote different genetic lines: blue: line C; red: line D; green: line E. Model fits are shown as continuous lines and the optimal values for parameters b, Cu, C1, and C2 are given in Table 4

The brute‐force approach to find the optimal parameter combination to fit all life‐history traits simultaneously provided an overview of the optimization landscape, which clearly showed a unimodal peak (Appendix S3). JMat = 1.8, C1 = 0.333, and Cu = 0.350 yielded the best fits, but note that model parameters interacted. Using slightly different values for Tref, JMat, C1, and Cu results in corresponding changes in estimated values for C2 and b. Model fits to the *D. magna* data generally resulted in good fits (Table 4), capable of capturing the nonlinearity in the data (Figure 6), especially at nonstressful temperatures. The model underestimated age at maturity and asymptote at temperatures above 26°C (Figure 6b,d). Fitting each genetic line separately resulted in parameter estimates that differed subtly between the three genetic lines, with the lowest value for b in genetic line E, giving rise to its larger size at asymptote and maturity (see Appendices S4 and S5).

**Table 4:** Parameter values resulting from optimization of the rate model to empirical data of *Daphnia magna*. Traits were fitted simultaneously for all genetic lines lumped and for each line separately. Model fits for all genetic lines are reported also when parameter b was fixed at 0.0 or C2 was fixed at 1.0. JMat = 1.8 and f2 = 0.8. *R* ^2^ show goodness of fit for each of the four traits (Vmax = Asymptotic size, AmaxV = Asymptotic age, Vmat = size at maturity, Amat = age at maturity). *R* ^2^ for fits on separate genetic lines give the goodness of fit for the three fits combined. ^F^ indicates value was fixed

Genetic line	*b*	Cu	C1	C2	SSres	*R* ^2^ for Vmax, AmaxV, Vmat, Amat
All	0.008	0.333	0.350	1.011	8.803	.57 + .64 + .21 + .57
	0.0^F^	0.333	0.333	1.017	14.256	.55 + .45 + .12 + .54
	0.008	0.333	0.383	1.0^F^	18.288	−.37 + .68 + .18 + .56
C	0.014	0.333	0.350	1.010		.71 + .58 + .28 + .64
D	0.006	0.333	0.350	1.013		
E	0.001	0.333	0.350	1.007		

Some parameters were fixed because they were not the focus of this study, but goodness of fit may correlate with the actual value at which these extra parameters were fixed. The reference temperature was fixed at 10°C, because this was the lowest tested temperature. A lower reference temperature of 9°C improved the fit of separate genetic lines as this allowed variation in the predicted size at 10°C. The fraction of uptake for soma (f2) was fixed at 0.8 (Table 1), and tests showed that higher or lower values for f2 did not improve model fits to data. Increasing this fraction increases energy allocation to growth plus maintenance and consequently increases body size and age at maturity, but had no or very small effects on age at asymptote (Appendix S2). Moreover, effects of b and C2 on size and age at a given stage remained similar for different values of f2, indicating that qualitative predictions (i.e., the patterns shown in Figure 5) are robust to such differences. The ability of the resource allocation model to fit the data on life‐history traits could in principle also depend on the calculation of those life‐history traits. Age and size at asymptote were both based on estimated von Bertalanffy growth curves, in which the von Bertalanffy growth parameter *K* was fixed at 0.046 across all temperatures, because it interacts with *V*_max_ (Pauly, 1979), which is the parameter we wanted to estimate for each temperature. The precise value of *K* = 0.046 was chosen because it resulted in the highest number of successful fits for individual *Daphnia* growth curves. While increasing or decreasing this value slightly altered the average estimated *V*_max_, it did not qualitatively alter the pattern (e.g., genetic line E still showed the strongest TSR; Appendix S1).

## 4 DISCUSSION

As it is increasingly unlikely that a single, general mechanism can explain the TSR, a more fruitful approach could be to consider multiple mechanisms, each with their own domain of applicability. We followed the suggestion by Forster and Hirst (2012) to examine temperature effects on size separately for different life stages. We observed that the strength of thermal responses differed between life stages and between genetic lines, demonstrating substantial variation in the form of the TSR even within the same species. Our results suggest that the physiological mechanisms governing size at maturity are distinct from those governing asymptotic size. These observations make it improbable that a single, general mechanism gives rise to the TSR.

We found that development rate has a stronger thermal dependency compared to that of growth and that differences across genetic lines in their thermal sensitivity of the ratio between growth rate and development rate corresponded to the pattern in size at maturity, with genetic line E having relatively high growth rates and large size at maturity. These results support the mechanism proposed to explain the TSR by van der Have and de Jong (1996). Other researchers have likewise found a temperature‐dependent shift in the ratio between somatic growth rate and development rate (Forster & Hirst, 2012; Walters & Hassall, 2006), suggesting that such decoupling is an important part of explaining the TSR (Forster, Hirst, & Atkinson, 2011), at least for size at maturity. For asymptotic size, differences in resource allocation do not matter (Figure 5), although they do affect age at asymptotic size.

Our results indicate that resource limitations become stronger at higher temperatures and larger body size, causing growth to decelerate at a smaller size at warmer temperatures (Appendix S6). Stronger limitation on growth in high temperatures was also indicated by the declining contribution of adult growth to asymptotic size in *D. magna* as temperatures rise (Appendix S6) and by different thermal reaction norms for growth in small and large animals (Figure 4). These results all suggest that there is an upper ceiling to the asymptotic size that can be attained and that this ceiling is raised in colder temperatures. A large asymptotic size in the low temperatures was not attained by fast adult growth but mainly by an extended growth period (Appendix S6). Similar patterns have been documented for other species like the isopod *Idotea baltica* and the fish *Pleurogrammus azonus* (Morita et al., 2015; Panov & McQueen, 1998; Strong & Daborn, 1980; Sutcliffe, Carrick, & Willoughby, 1981). This suggests that the effects on growth depend on interactive effects between temperature and body size in accordance with the notion that resources become more limited at larger body sizes and warmer conditions.

Given that both food was supplied ad libitum and all test vials had continuous contact with air, neither food nor oxygen limitation would appear likely. However, resource supply needs to be viewed in the context of resource demand and it could be that limitations arise from capacity becoming insufficient to meet the temperature‐induced increase in demand. Indeed, Hanazato and Dodson (1995) demonstrated stronger size reductions in *Daphnia pulex* when reared under low oxygen conditions at 21°C. Their study did not include different temperatures, but we previously showed for the crustacean *Asellus aquaticus* that growth was constrained especially under warm and hypoxic conditions, suggesting that oxygen limitation is a prerequisite for the TSR to manifest (Hoefnagel & Verberk, 2015), even in an animal that is quite tolerant to heat and hypoxia (Hervant, Mathieu, & Messana, 1998; Verberk, Leuven, van der Velde, & Gabel, [Link]).

An increase in mortality risks with temperature has been suggested as an ultimate cause for maturation of animals at a smaller size under warm conditions (Kozłowski et al., 2004). Angilletta et al. (2004) concluded that the thermal sensitivity of mortality was too low to explain the TSR, but acknowledges that published mortality rates in a laboratory setting may underestimate those that occur in the field where competition and predation are also contributing, sometimes synergistically, to aggravate mortality. An increase in mortality with temperature could act as a selection pressure, strengthening the TSR not only at maturity but also at asymptote. Investments in growth generate a future return as larger individuals have a greater fecundity. But these future returns are contingent upon the survival of the individual. In this respect, it is interesting to note that the genetic line with the highest mortality, line E, also exhibited the strongest TSR at asymptote. In addition to mortality risks acting as a selection pressure, the time needed to grow to asymptotic size depends on resources being allocated to growth and slow growers may simply not reach asymptotic size within their typical lifespan.

While our data give a quantitative view of the phenotypical effects of temperature, the underlying physiological mechanisms could be disentangled with the resource allocation model. The resource allocation model served to get a qualitative view of the contribution of different mechanisms to the TSR. Our resource allocation model could mimic the mechanisms featuring in the two different explanations, with temperature‐dependent allocation (the b parameter) affecting size at maturity, but not asymptotic size (Figure 5a), while a steeper temperature dependence of the maintenance rate compared to uptake rate (the C2 parameter) gives rise to resource limitations at asymptotic size (Figure 5b). When excluding the allocation mechanism, by fixing parameter b at 0.0, we could still obtain reasonably good fits as increased values of C2 could somewhat compensate to generate comparable fits for size at maturity (but not age at maturity) (Table 4). However, fits were poor when parameter C2 was fixed at 1.0, especially for asymptotic size, indicating that the difference in thermal sensitivity between uptake and maintenance expressed by C2 was essential for generating thermal responses in asymptotic size in our model. The estimated values for C2 of 1.014 and 1.017 (when b is fixed to 0) corresponded to Q_10_ values for maintenance of 2.30 and 2.37, which are within the common range for biological rates (Daoud et al., 2007).

In our model, growing animals gradually decrease allocation to growth, culminating in allocating all surplus energy toward reproduction when they reach asymptotic size. This is in contrast to other life‐history models that often include a sharp switch at the moment of maturity (see, e.g., Brunel, Ernande, Mollet, & Rijnsdorp, 2013 and Ohnishi, Yamakawa, Okamura, & Akamine, 2012). It has been argued that models based on mathematical equations that describe a growth curve phenomenologically such as that of von Bertalanffy (1960) are unsuitable for modeling age and size at maturity when such a switch is not included (Czarnoleski & Kozłowski, 1998; Day & Taylor, 1997). However, such a switch proved not essential for generating the TSR and accurately fitting the life history of *D. magna*. The idea that reproduction results from a transition in allocation from development to reproduction and additionally a gradual decrease in allocation toward growth, rather than a switch from growth to reproduction, was inspired by dynamic energy budget (DEB) theory (Kooijman, 2000). An important difference is that our model allows for variable thermal dependencies, where DEB theory assumes equal thermal dependence of all physiological rates within an organism (Kooijman, 2000, p. 57).

A logical consequence of our model whereby animals allocate all surplus energy toward reproduction when they reach asymptotic size is that the ratio between resources allocated to growth on the one hand and those allocated to either development or reproduction on the other hand necessarily changes with size, making it difficult to completely disentangle the effect of resource limitation from allocation, even when the parameter b is fixed at 0.0. Fixing b at 0.0 did set the allocation ratio between development and growth to a constant value at 0.5 × Vmax across temperatures, but parameter changes resulting in differences in asymptotic size will be accompanied by differences in allocation ratios for a given size. Therefore, thermal responses in size at maturity can also result from resource limitations (i.e., C2 > 1; open squares in Figure 5a). However, age and size at maturity were best predicted by a model whereby b > 0, suggesting that temperature‐dependent allocation plays an important role for the TSR at maturity.

The resource allocation model gave different parameter estimates for the genetic lines, reflecting the observed differences in growth and development rates between genetic lines. Genetic line E, which grew fastest and to the largest size, also had the lowest estimate for parameter b, which means that a higher resource allocation to growth was maintained under warm conditions. This shows that subtle differences within and between populations can be captured with our resource allocation model and that phenotypic variation may be coupled to variation in physiological rates.

Fitting all four life‐history traits in one optimization considerably reduced respective R^2^s (Table 4) relative to allowing different parameter values for each trait, suggesting that either the optimal set of parameter values went undetected or there is no single set of parameter values that can adequately fit the traits at maturity and those at asymptote simultaneously. This suggests that physiological rates change during the lifetime of *D. magna*, possibly such that the thermal sensitivity of rates itself depends on body size, that is, mass exponents differ with temperature (see also Kozłowski et al., 2004; Verberk & Atkinson, 2013). Such temperature dependency of mass exponents has been reported for metabolism (Carey & Sigwart, 2014; Killen, Atkinson, & Glazier, 2010) and could explain why growth efficiency tends to increase rather than decrease with temperature (see Angilletta & Dunham, 2003).

Before applying this model and our conclusions to other species than *D. magna*, it must be taken into account that the resource allocation model was explicitly designed to fit an indeterminate grower which reaches maturity before asymptotic size. Another consideration is that of manner of growth. *D. magna* grows via many molts with a relatively small molt increment and continues to grow after reaching maturity. As a consequence, size at maturity and final asymptotic size are distinct size metrics and both our empirical results and resource allocation model suggest that distinct mechanisms are involved in shaping the respective forms of the TSR. Our model and conclusions could be extended and applied to other indeterminate growers that reach maturity before asymptotic size. For determinate growers, a different model structure may be needed. For example, in most holometabolous insects, final size and size at maturity are identical. The gastropod *Monetaria annulus* obeys the TSR and also shows determinate growth, but matures *after* the final size has been reached (Irie, Morimoto, & Fischer, 2013). Still, in the determinate growers, similar mechanisms may cause a TSR in those species. For example, Kutcherov, Lopatina, and Kipyatkov (2011) found different thermal dependencies of growth and development rates in the beetle *Chrysomela populi*.

## 5 CONCLUSION

In conclusion, the widespread observation that animals are smaller in warm environments than in cold environments needs to be contextualized and considered separately for different life stages. Temperature‐size responses manifested at maturity differ from those manifested at asymptotic size and from the transgenerational effects on offspring size. We provide evidence that thermal shifts in allocation of resources may completely or partly give rise to thermal plasticity in size at maturity, whereas thermal plasticity in asymptotic size is more likely governed by temperature‐induced resource limitations. Different mechanisms may act in concert, and by combining these mechanisms into a single resource allocation model, we could isolate the distinct underlying mechanisms for the different forms of the TSR. Such models therefore provide a promising tool for answering a long‐standing question and illustrate the need to explicitly consider the size measure that is studied. We encourage researchers to include age‐at‐size and growth rate in their analyses, because they help in defining the temperature‐size rule more precisely and could aid in clarifying and explaining observed thermal size responses.

## CONFLICT OF INTEREST

None declared.

## AUTHOR CONTRIBUTIONS

KNH and EHJV collected the data in the laboratory, EJ and WCEPV designed the experiment, KNH and WCEPV analyzed the data, and all authors contributed to the development of the resource allocation model. All authors contributed to the drafts and approved it for publication.
