# Supplementary material for: The temperature‐size rule in Daphnia magna across different genetic lines and ontogenetic stages: Multiple patterns and mechanisms
Source: Ecol Evol. 2018 Mar 13;8(8):3828–41. doi: 10.1002/ece3.3933 (PMC5916275; doi:10.1002/ece3.3933)
Supplement: Supplementary file 1 [file ECE3-8-3828-s001.docx]

**Appendix S1**. Effect of fixing parameter K.

K is a parameter in the Von Bertalanffy growth function that determines the rate at which the asymptote (Vmax) is approached.

Values within the range of K=0.026 until K=0.066 result in R^2^ for all Bertalanffy growth curves > 0.85, and estimates for Vmax vary slightly within this range, indicating that fixing K does not greatly impair the fitting of Bertalanffy growth function to individual growth data.


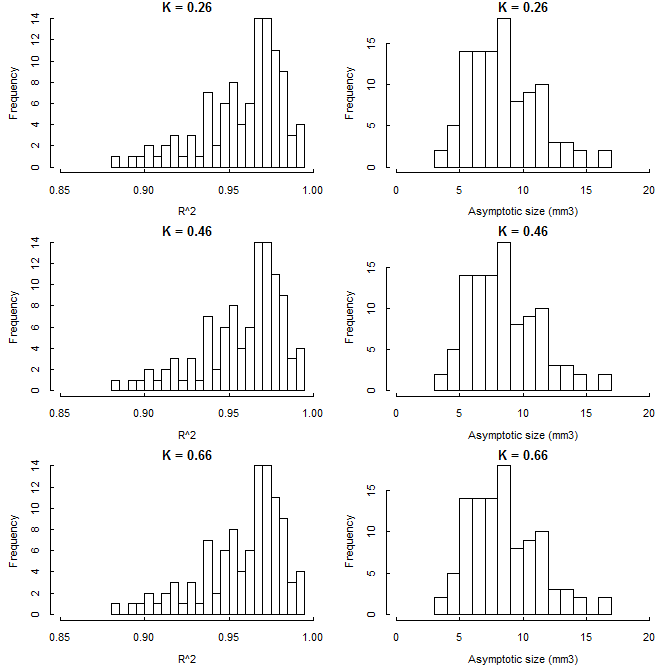


**Appendix S2**. Effect of f2 (fraction to soma) on model predictions.

The fraction of energy uptake that is allocated to soma (growth + maintenance) was fixed at 0.7 (top row), 0.8 (middle row) or 0.9 (bottom row). Its value affects absolute values for the life-history traits, but does not change the pattern. The values for other parameters were kept constant during this model check (b = 0, Cu = 0.4, C1 = 0.34, C2 = 1.02, JMat = 1.7, J2g = 967.5). Note the differences in y-axis scaling.
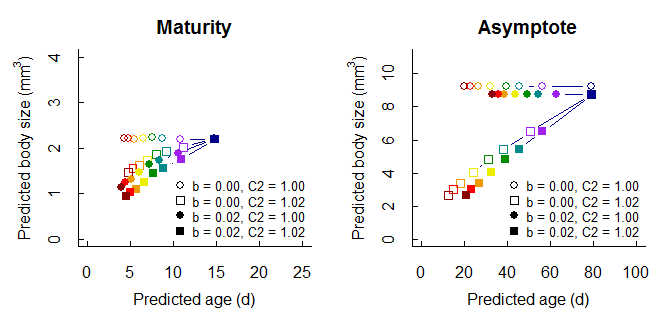


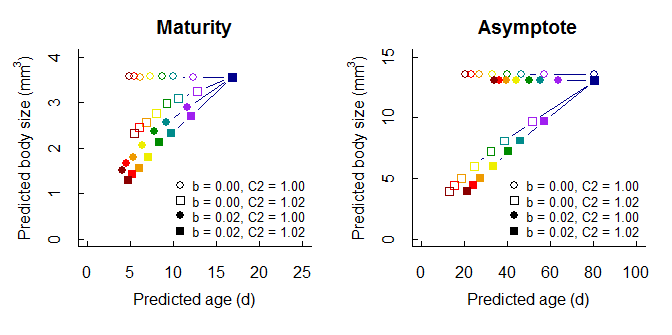


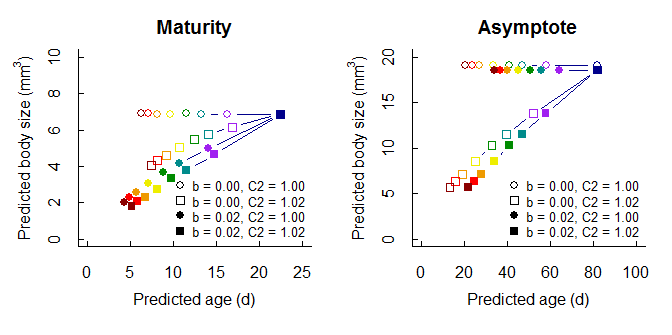


**Appendix S3**. Results of brute-force optimization.

Below we calculated the combined sum of squared residuals for all four life-history traits. We tested a range of values for all parameters. Combined SSres was calculated as

(SSVmax-SSres1)/(SStot1-SSres1)*10 + (SSAmaxV-SSres2)/(SStot2-SSres2)*10 +

(SVmat-SSres3)/(SStot3-SSres3)*10 + (SSAmat-SSres4)/(SStot4-SSres4)*10,

Where SS_trait is the realized SSres for each trait, SStot_n (where n is between 1 and 4) is the maximum allowed sum of squares (model equals a horizontal line through the average value) for each trait and SSres_n is the sum of squares in case of a perfect fit through the averages at each test temperature for each trait. This method scales combined SSres from 0 (perfect fit) to 40 (maximum allowed SSres). Grey dots indicate combined SSRes > 40. The tested ranges are b = 0.00 – 0.03; Cu = 0.3 - 0.5; C1 = 0.3 - 0.4; C2 = 1.00 – 1.04, resolution b and C2 = 20 steps, Cu and C1 = 10 steps, JMat = 1.6, 1.7, 1.8, 1.9, 2.0. Lowest SSres = 7.96, was found at Cu = 0.333, C1 = 0.350, b = 0.008, C2 = 1.011 and JMat = 1.8.


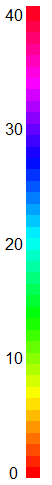

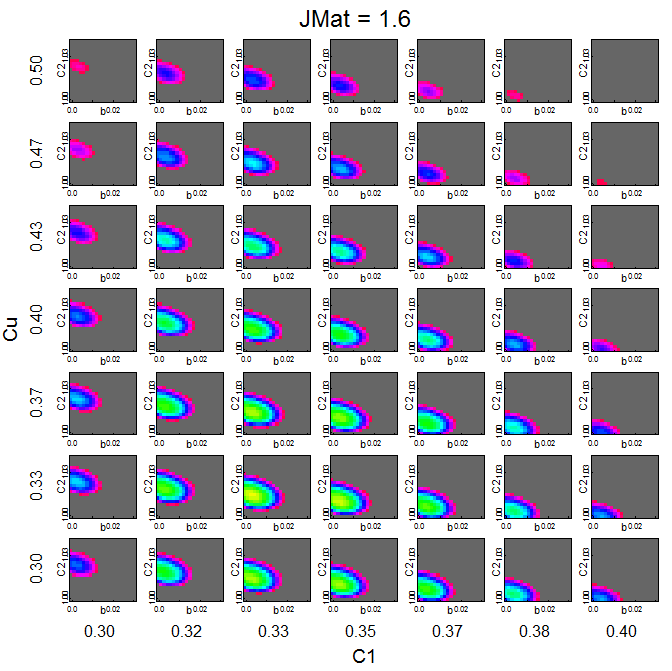


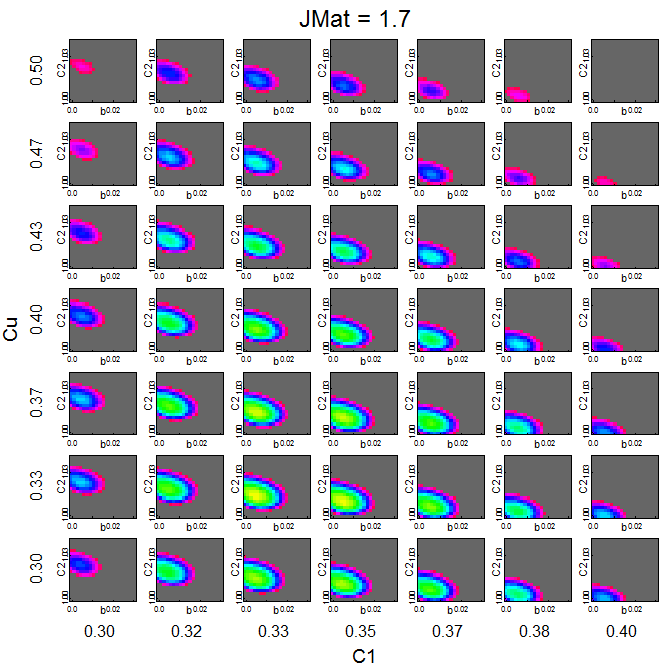


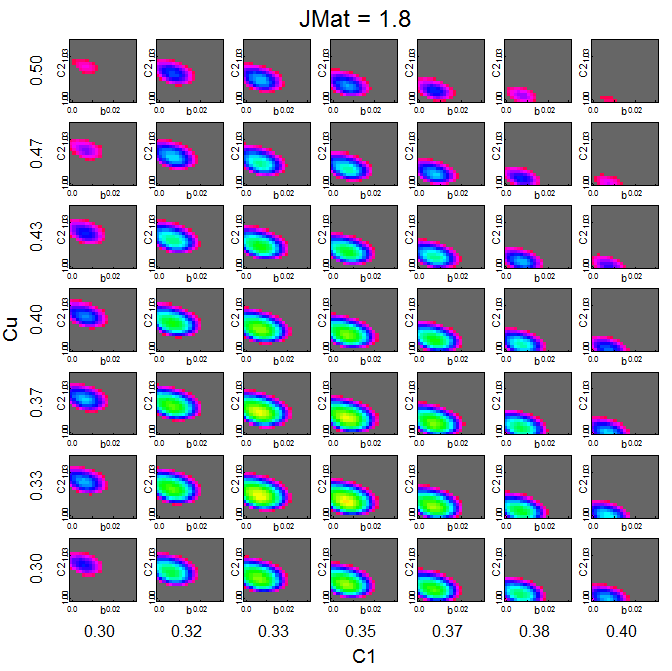


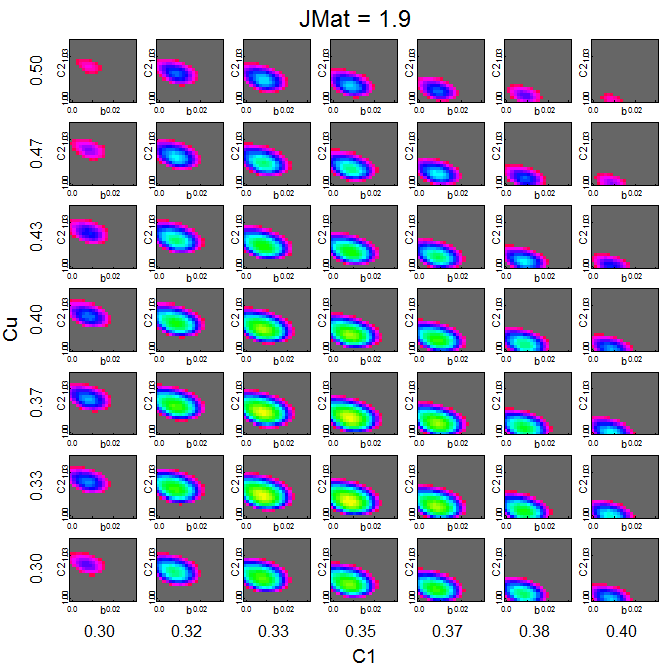


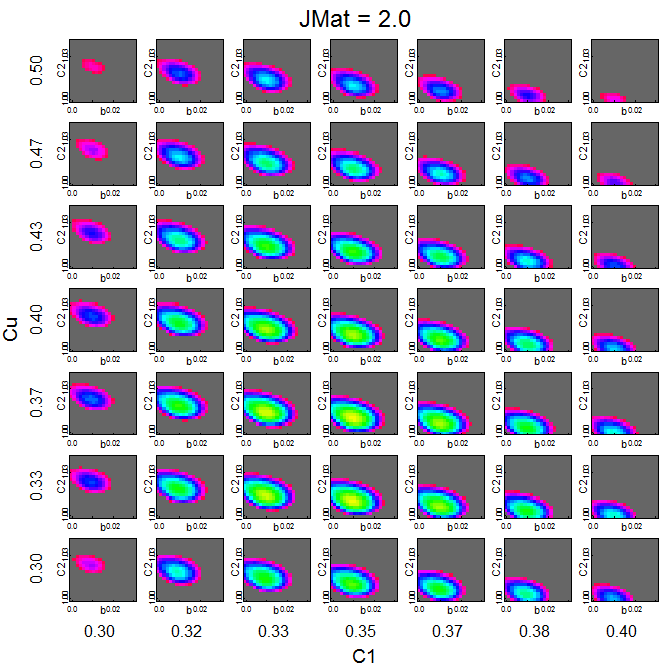


**Appendix S4**. Model simulation for genetic lines when all traits are fitted simultaneously. The first panel shows the Sum of squared residuals (SSres) for all values of b and C2 at JMat = 1.7, Cu = 0.333 and C1 = 0.333, f2 = 0.8 and Tref = 10. The other panels show the landscape of SSres when fitted for data on genetic lines separately. The best fit differs between genetic lines. Colours denote SSres, where grey means SSres > 40 and brighter yellow means lower values.


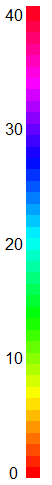

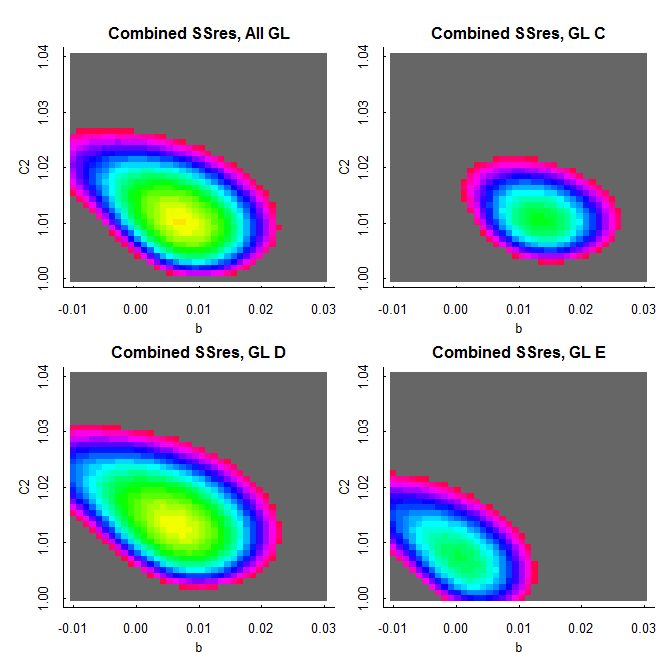


**Appendix S5**. Model predictions for genetic lines separately. Model parameters b and C2 were allowed to vary between genetic lines, so that the intercept is constant, but the slope of the curves can vary. Colours represent genetic line C (blue), D (red) and E (green). Parameter values as in Table 6.


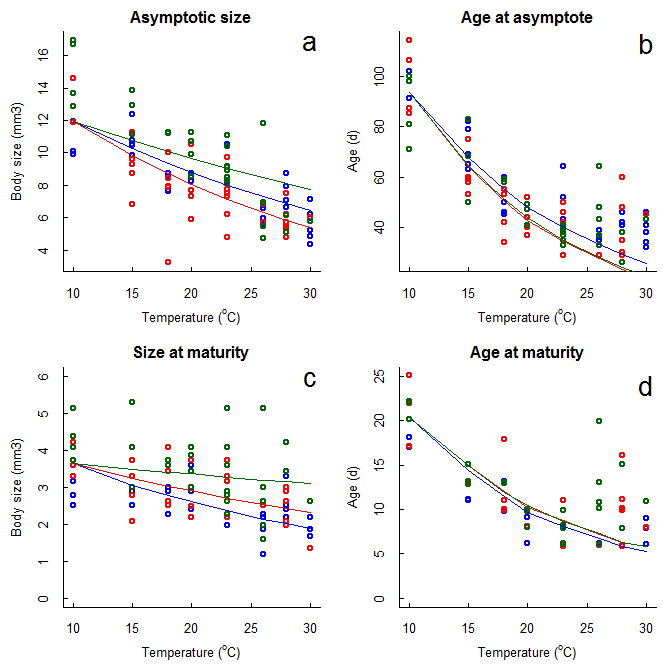


**Appendix S6**. Effect of temperature on adult growth and maximum growth rate.


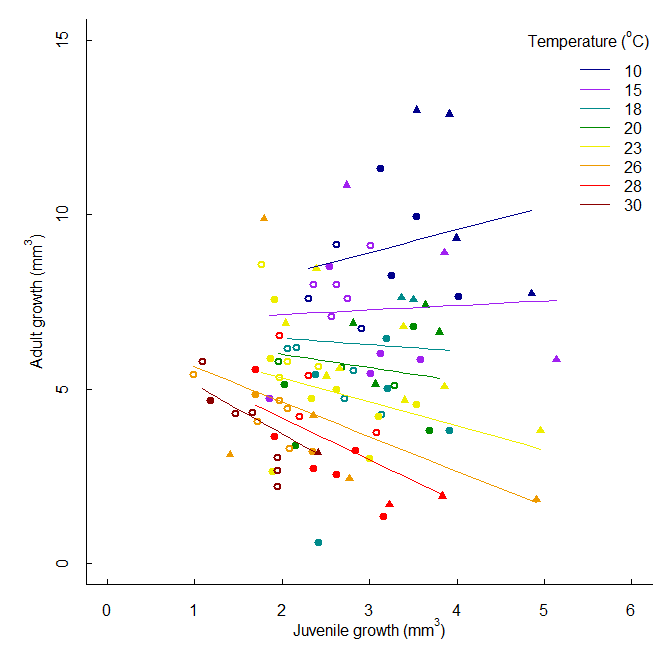


Figure S6.1. Relation between juvenile growth and adult growth in Daphnia magna based on individual Von Bertalanffy growth curves. Coloured lines are based on statistical model (Table S6.1). Colours denote temperatures, and open circles, filled circles, and triangles denote genetic line C, D, and E, respectively. Individual asymptotic size (and adult growth) was estimated using the Von Bertalanffy growth function (Eq. 2).

Table S6.1. Summary table of linear model for juvenile and adult growth in Daphnia magna (Fig. S6.1).

|  | Source | Estimate | Std. Error | t value | DF | P |  |
| --- | --- | --- | --- | --- | --- | --- | --- |
| Adult growth | (Intercept) | 7.22462 | 2.37168 | 3.046 | 83 | 0.0030 | ** |
| R^2^ = 0.53 | Juvenile growth | 1.66324 | 0.74496 | 2.233 |  | 0.0279 | * |
|  | Temperature | -0.02315 | 0.09846 | -0.235 |  | 0.8146 |  |
|  | Juvenile growth : T | -0.10223 | 0.03280 | -3.117 |  | 0.0024 | ** |
|  |  |  |  |  |  |  |  |
| Asymptotic size | (Intercept) | 14.074676 | 1.011303 | 13.917 | 96 | <0.001 | *** |
| R^2^ = 0.60 | Adult lifespan | 0.020739 | 0.009273 | 2.237 |  | 0.0276 | * |
|  | Temperature | -0.291301 | 0.037202 | -7.830 |  | <0.001 | *** |


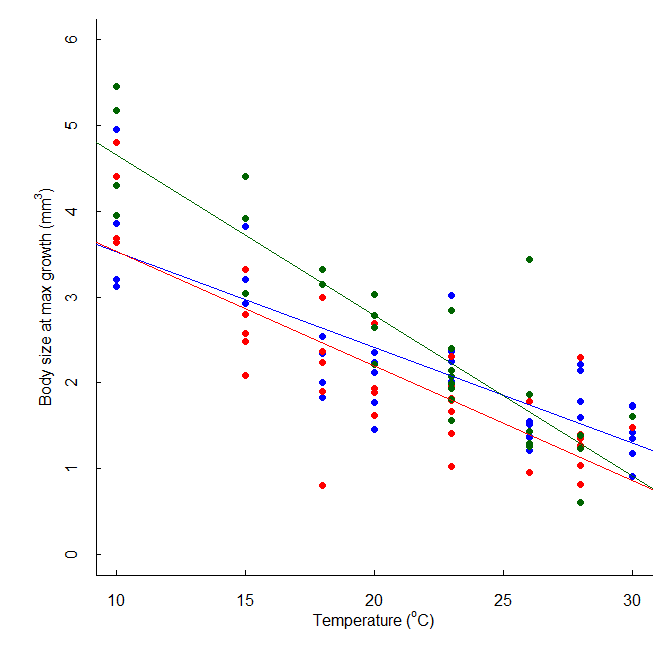


Figure S6.2. Thermal reaction norm of the size at which the growth rate was maximised in Daphnia magna. Data are based on individual-based Von Bertalanffy growth curves and lines represent linear model fits (Table S6.2). Colours denote different genetic lines: Blue: line C; Red: line D; Green: line E.

Table S6.2. ANOVA table (type 3 sum of squares) of linear models on size at maximum growth rate over temperature in Daphnia magna.

| Source | Sum of squares | DF | F-value | P-value |  |
| --- | --- | --- | --- | --- | --- |
| (Intercept) | 65.956 | 1 | 225.3520 | < 2.2e-16 | *** |
| Temperature | 19.422 | 1 | 66.3586 | 1.237e-12 | *** |
| GenLine | 4.941 | 2 | 8.4406 | 0.0004148 | *** |
| T:GenLine | 3.506 | 2 | 5.9901 | 0.0035128 | ** |
| Residuals | 28.683 | 98 |  |  |  |

**Appendix S7**. Model selection for growth rate and development rate.

Tested models are given for model selection based on AIC values. Genetic line and temperature are always present as main effect. Differences between models are in interactions between genetic line and temperature. For both tables, the model with the lowest AIC value is used in the main text and presented in Fig. 3 and Table 3. All models are run with the function lm() in R. : indicates interaction.

Table S7.1 Models for maximum growth rate.

| Model | AIC | ΔAIC |
| --- | --- | --- |
| GenLine + T + T^2^ + T^3^ + Genline:T^2^ | -549.9 | 0.0 |
| GenLine + T + T^2^ + T^3^ + GenLine:T + GenLine:T^2^ | -549.6 | 0.3 |
| GenLine + T + T^2^ + T^3^ | -548.7 | 1.2 |
| GenLine + T + T^2^ + T^3^ + GenLine:T + GenLine:T^2^ + GenLine:T^3^ | -548.7 | 1.2 |
| GenLine + T + T^2^ + T^3^ + Genline:T | -548.6 | 1.3 |

Table S7.2 Models for development rate.

| Model | AIC | ΔAIC |
| --- | --- | --- |
| GenLine + T + T^2^ + T^3^ + Genline:T^2^ | -810.1 | 0.0 |
| GenLine + T + T^2^ + T^3^ + GenLine:T + GenLine:T^2^ | -809.4 | 0.7 |
| GenLine + T + T^2^ + T^3^ + Genline:T | -808.7 | 1.4 |
| GenLine + T + T^2^ + T^3^ | -808.1 | 2.0 |
| GenLine + T + T^2^ + T^3^ + GenLine:T + GenLine:T^2^ + GenLine:T^3^ | -806.8 | 3.3 |

**Appendix S8**. Model fits of size over temperature with log-transformed size.


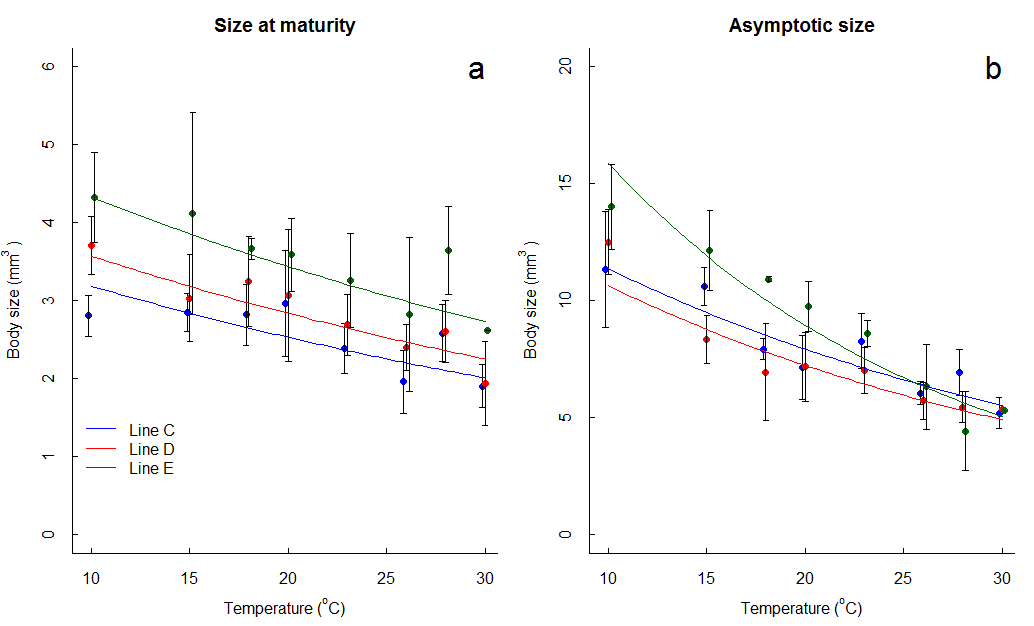


Figure S8.1. Thermal reaction norms for size in *Daphnia magna*. Size (in mm^3^) at maturity (a) and at asymptote (b) are given for each temperature and genetic line. Asymptotic body size was estimated using the Von Bertalanffy growth function (Eq. 2). Colours denote different genetic lines: Blue: line C; Red: line D; Green: line E. Details of the fitted lines are given in Table S8.1. Error bars denote 95% confidence intervals of the data to indicate statistical differences.

Table S8.1. ANOVA tables (type 3 sum of squares) of log-linear models for two size measures in *Daphnia magna* based on temperature (T) and genetic line (GenLine), and their interaction. Note the different number of observations for the two size measures.

| Trait | Source | Sum of Squares | Df | F-value | P-value |  |
| --- | --- | --- | --- | --- | --- | --- |
| ln(Size at maturity) | (Intercept) | 13.4833 | 1 | 280.078 | <2.2e-16 | *** |
|  | Temperature | 2.1772 | 1 | 45.224 | 8.658e-10 | *** |
|  | GenLine | 1.6486 | 2 | 17.123 | 3.474e-07 | *** |
|  | Residuals | 5.1993 | 108 |  |  |  |
| ln(Asymptotic size) |  |  |  |  |  |  |
|  | (Intercept) | 23.8159 | 1 | 546.3369 | <2.2e-16 | *** |
|  | Temperature | 2.0316 | 1 | 46.6052 | 7.302e-10 | *** |
|  | GenLine | 0.4920 | 2 | 5.6437 | 0.004788 | ** |
|  | T : GenLine | 0.3027 | 2 | 3.4725 | 0.034909 | * |
|  | Residuals | 4.2720 | 98 |  |  |  |
