# Supplementary figures and images for: The temperature‐size rule in Daphnia magna across different genetic lines and ontogenetic stages: Multiple patterns and mechanisms
Source: Ecol Evol. 2018 Mar 13;8(8):3828–41. doi: 10.1002/ece3.3933 (PMC5916275; doi:10.1002/ece3.3933)

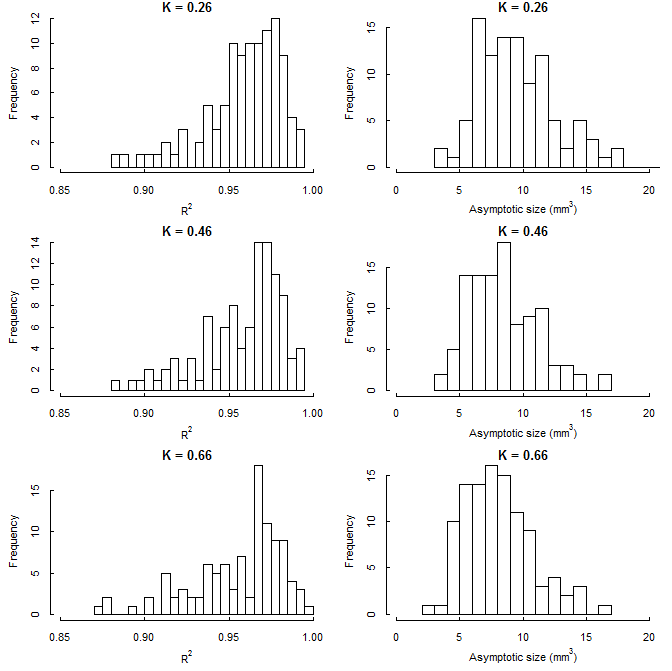

Supplement: Supplementary file 2 [file ECE3-8-3828-s002.tiff]

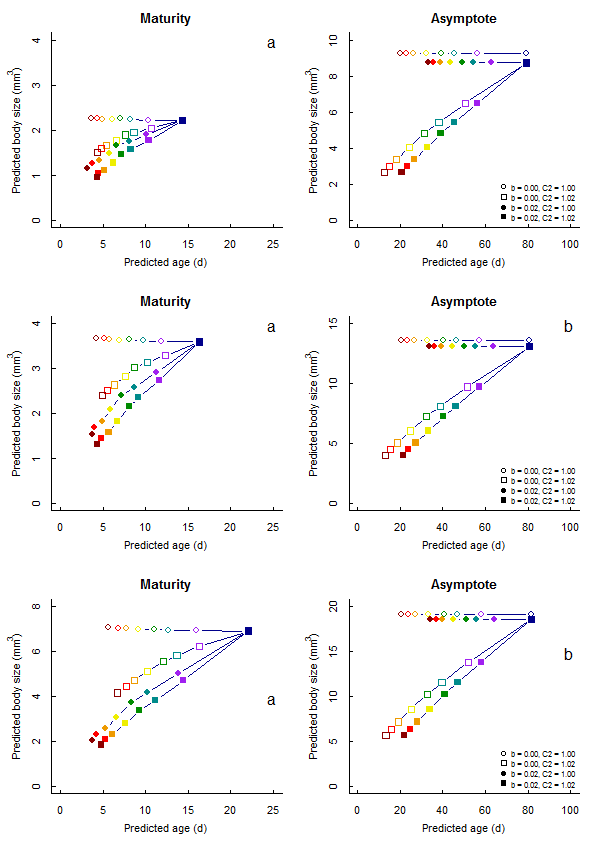

Supplement: Supplementary file 3 [file ECE3-8-3828-s003.tiff]

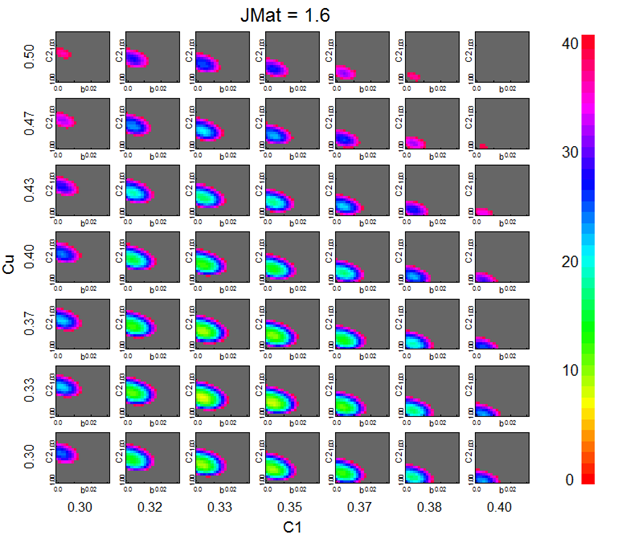

Supplement: Supplementary file 4 [file ECE3-8-3828-s004.tif]

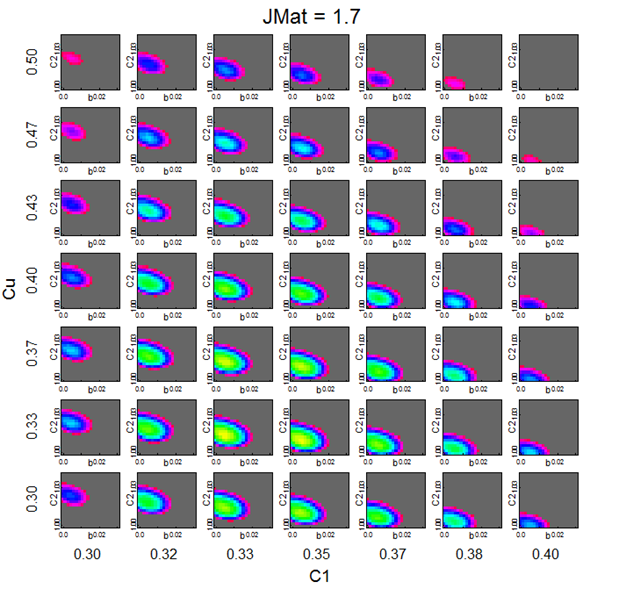

Supplement: Supplementary file 5 [file ECE3-8-3828-s005.tif]

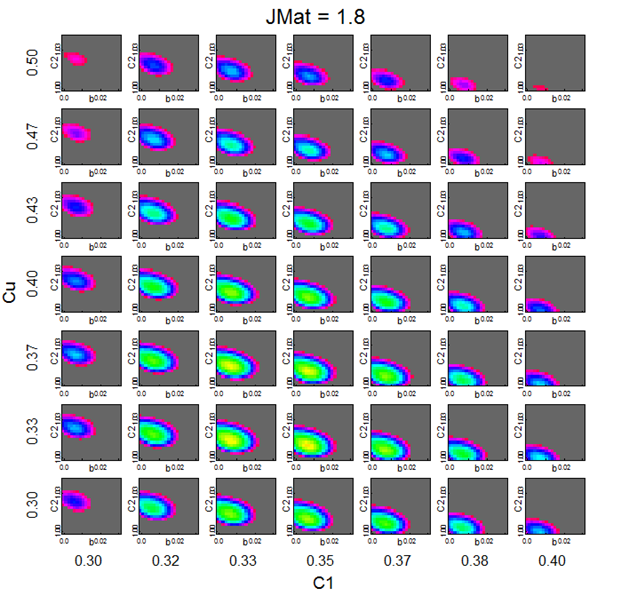

Supplement: Supplementary file 6 [file ECE3-8-3828-s006.tif]

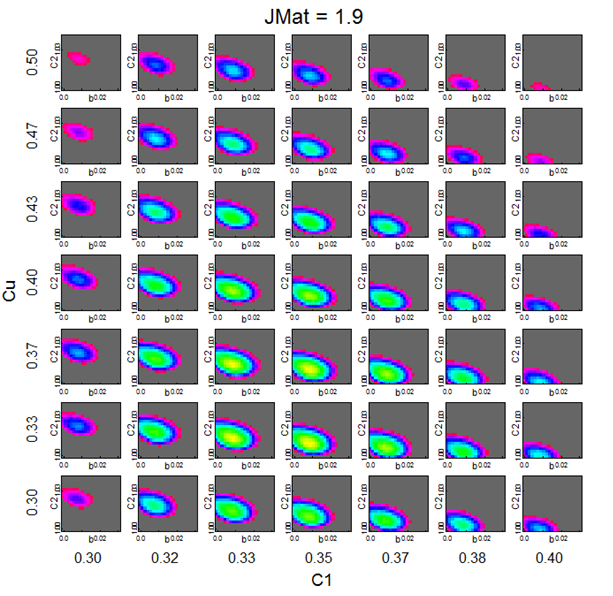

Supplement: Supplementary file 7 [file ECE3-8-3828-s007.tif]

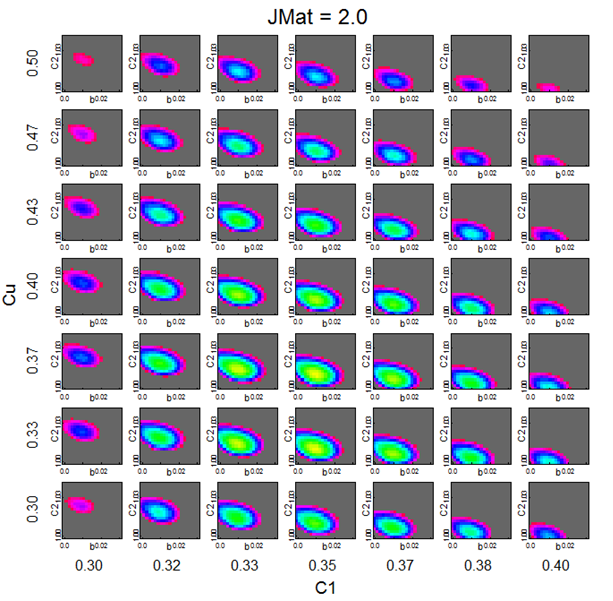

Supplement: Supplementary file 8 [file ECE3-8-3828-s008.tif]

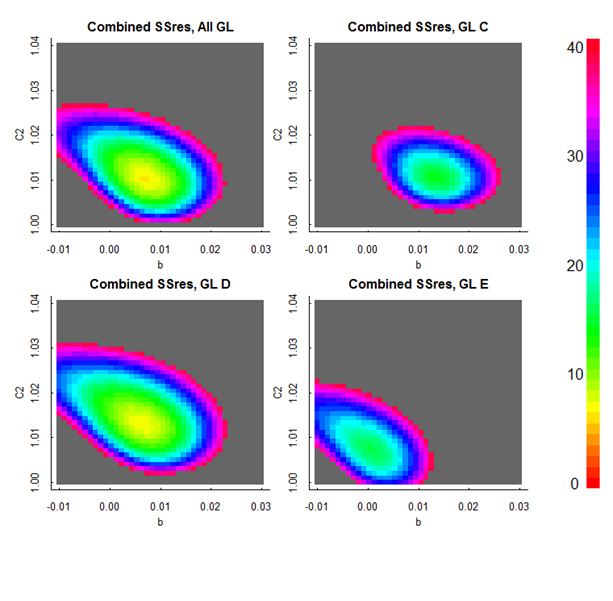

Supplement: Supplementary file 9 [file ECE3-8-3828-s009.tif]

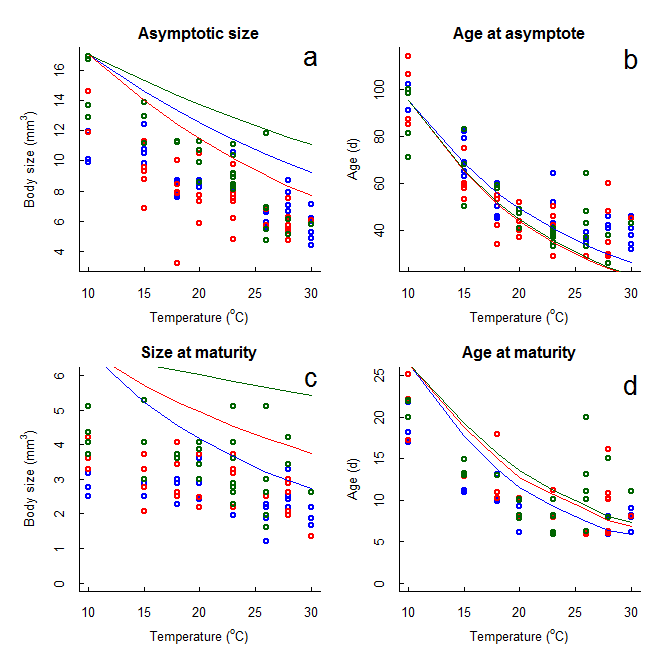

Supplement: Supplementary file 10 [file ECE3-8-3828-s010.tiff]

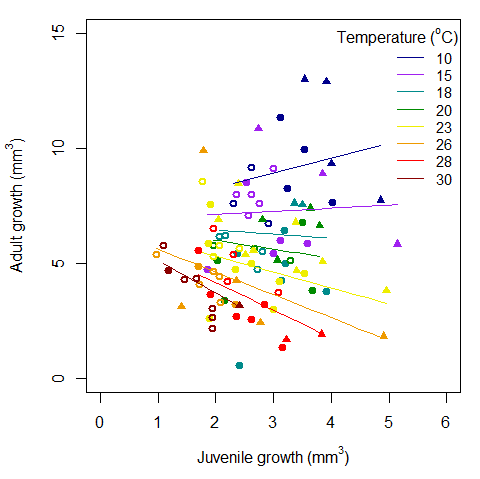

Supplement: Supplementary file 11 [file ECE3-8-3828-s011.tiff]

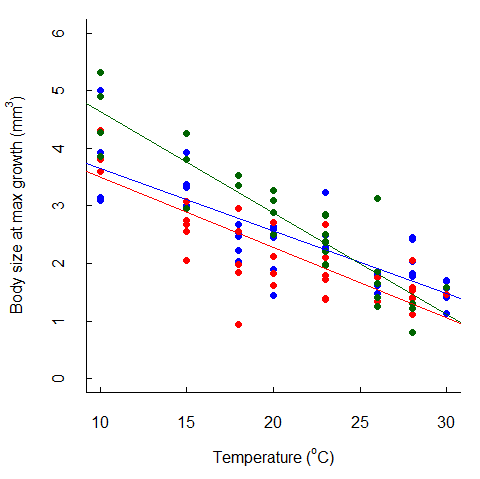

Supplement: Supplementary file 12 [file ECE3-8-3828-s012.tiff]

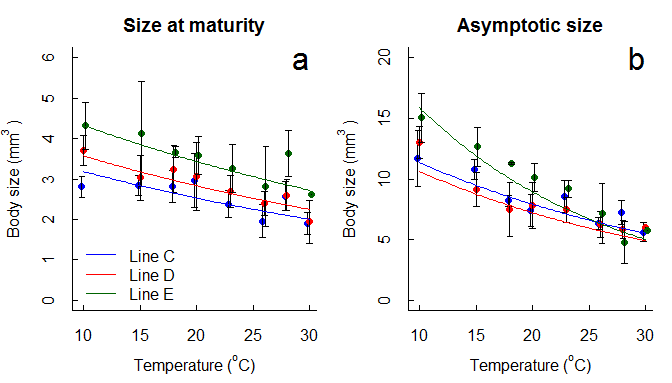

Supplement: Supplementary file 13 [file ECE3-8-3828-s013.tiff]
